# An Introduction to Software Tools, Data, and Services for Geospatial Analysis of Stroke Services

**DOI:** 10.3389/fneur.2019.00743

**Published:** 2019-08-07

**Authors:** Mark Padgham, Geoff Boeing, David Cooley, Nicholas Tierney, Michael Sumner, Thanh G. Phan, Richard Beare

**Affiliations:** ^1^Active Transport Futures, Muenster, Germany; ^2^School of Public Policy and Urban Affairs, Northeastern University, Boston, MA, United States; ^3^Symbolix Pty Ltd, Melbourne, VIC, Australia; ^4^Department of Econometrics and Business Statistics, Monash University, Melbourne, VIC, Australia; ^5^Australian Antarctic Division, Department of the Environment and Energy, Kingston, TAS, Australia; ^6^Clinical Trials Imaging and Informatics Division of Stroke and Aging Research Group, Monash University, Melbourne, VIC, Australia; ^7^Stroke Unit, Monash Medical Centre, Melbourne, VIC, Australia; ^8^Department of Medicine, Monash University, Melbourne, VIC, Australia; ^9^Developmental Imaging, Murdoch Children's Research Institute, Melbourne, VIC, Australia

**Keywords:** geospatial, software, data, review, stroke, emergency clot retrieval

## Abstract

**Background:** There is interest in the use geospatial data for development of acute stroke services given the importance of timely access to acute reperfusion therapy. This paper aims to introduce clinicians and citizen scientists to the possibilities offered by open source softwares (R and Python) for analyzing geospatial data. It is hoped that this introduction will stimulate interest in the field as well as generate ideas for improving stroke services.

**Method:** Instructions on installation of libraries for R and Python, source codes and links to census data are provided in a notebook format to enhance experience with running the software. The code illustrates different aspects of using geospatial analysis: (1) creation of choropleth (thematic) map which depicts estimate of stroke cases per post codes; (2) use of map to help define service regions for rehabilitation after stroke.

**Results:** Choropleth map showing estimate of stroke per post codes and service boundary map for rehabilitation after stroke. Conclusions The examples in this article illustrate the use of a range of components that underpin geospatial analysis. By providing an accessible introduction to these areas, clinicians and researchers can create code to answer clinically relevant questions on topics such as service delivery and service demand.

## 1. Introduction

Endovascular clot retrieval (ECR) and thrombolysis enables reperfusion following ischemic stroke and results in dramatic reversal of neurological deficit in selected patients (1–6). The publication of the DEFUSE3 (Endovascular Therapy Following Imaging Evaluation for Ischemic Stroke) and DAWN (DWI or CTP Assessment with Clinical Mismatch in the Triage of Wake-Up and Late Presenting Strokes Undergoing Neurointervention with Trevo) trials potentially pushed the boundary for performing ECR in a small set of patients to 24 h (7, 8) and thrombolysis to 9 h (6). ECR treatment requires specialist centers with 24 h staffing by skilled stroke teams and interventional radiologists with unrestricted access to angiography suites. Key decisions for governments and policy makers include: how many centers are required to service a specified area, how to identify redundant centers, where should the centers be placed and what is the expected load on the centers (9). Many factors influence the final choice, including the availability of trained interventional neuroradiologists (INR), the number of cases required for INR to retain skills, and costs.

Related to the above considerations is the issue of transport model. One proposed model is direct transport to mothership (ECR hub) whereby ambulance bypass the smaller hospitals including those which are thrombolysis capable and take the patient to directly to the ECR hub (10, 11). The alternative model is the “drip and ship” model whereby patients are brought to the nearest thrombolysis center for advanced imaging and trigging on the need for thrombolytic drug and or ECR. A drawback to the drip and ship model is that there is an additional 99 minutes delay related to a second transfer of the case with large vessel occlusion (LVO) to the mothership (12). Investigators recently proposed inclusion of speed of delivery of thrombolysis as an additional consideration (11). Identification of patients for ECR at the pre-hospital level requires either the use of LVO scale or mobile stroke unit (MSU). Before LVO scales can be used in the field, the impact from its use on ECR hub loading (handling of large volume of ECR and non-ECR cases) has to be tested.

In the writing of this introduction, we have asked several data scientists (software engineers) to contribute the introduction to geospatial analysis for clinicians and other citizen scientists aspiring to work in the field. Alternatively, a deeper understanding of these geospatial methods will enable clinicians to collaborate with other researchers or citizen scientists on models to improve local stroke service. Both codes and data are provided so that once *R* and *Python* softwares are installed, the scientist can copy and paste the example codes to test run. These softwares typically involve relatively steep learning curves and as such instructions for running the softwares are provided; please see the more extensive codes and data on our github address https://richardbeare.github.io/GeospatialStroke/. The examples provided here are not exhaustive and are intended to stimulate creative use of these geospatial tools. The two examples we present are a choropleth (thematic map) and a service catchment basin estimation. A choropleth is a thematic map display in which regions are colored by a measure of the region. We use demographic and boundary data from the Australian Bureau of Statistics and incidence data from the NEMESIS (13, 14) study to estimate stroke cases per postcode and display the result on an interactive map. The service catchment basin estimation involves a Monte-Carlo simulation of patients attending a rehabilitation service of 3 hospitals. The catchment basin of each hospital is the region that has lower travel time to that hospital than any other. Catchment basins can be combined with incidence data to estimate load on rehabilitation centers. The data can be used to explore scenarios, such as the removal or addition of service centers.

## 2. Geospatial Analysis

*Geospatial analysis* or modeling of spatial data has traditionally been the domain of *geographic information systems (GIS)* specialists, employing commercial software and data products. Recent years, however, have seen the development of open source tools and free or low cost web services, such as Google Maps, that make geospatial analysis accessible and feasible to the non-specialist citizen scientist. In this article we introduce a family of computational techniques and services, collectively termed geospatial analysis tools, that can be applied to a range of questions relevant to stroke services. The codes used here are for two free and open source software environments—*R* and *Python*. Geospatial analysis can also be performed with Matlab (10); this software is not free and will not be discussed further. Geospatial analysis tools allow manipulation and modeling of geospatial data. These tools, data, and modeling techniques have a long track record in the quantitative geography, city and regional planning, and civil engineering research literatures. Geospatial data, in the context of stroke research, includes the location of patients and treatment centers, routes through the road network linking patients to treatment centers, geographic and administrative region boundaries (e.g., post codes, government areas, national boundaries) and disease incidence and demographic information associated with such regions.

### 2.1. Geospatial Frameworks

In geospatial analysis, the location of a data point on the Earth's surface is referred to in terms of longitude and latitude. In practice, longitude is the X axis and latitude is the Y axis. More complex data, such as national boundaries or administrative or postcode boundaries consist of sets of points connected together in defined orders, typically to produce a closed shape. Other structures, such as road networks, are also constructed using sets of points and include other types of information, such as speed limits, travel direction etc. A geospatial framework provides mechanisms for representing, loading, and saving geospatial data and performing fundamental mathematical operations. For example, the simple features (sf) (15) package, on which our R examples are based, provides structures to represent all manner of shapes and associate them with non-spatial quantities, perform transforms between coordinate systems, display shapes, compute geometric quantities like areas and distances and perform operations like intersections and unions. The equivalent Python framework is the geopandas package that provides a geospatial extension to standard data frames. A key emerging subdomain of geospatial analysis is spatial network analysis. Several open-source packages now exist for modeling and analyzing spatial networks, such as urban street networks, including dodgr for R (16) and OSMnx for Python (17).

### 2.2. Sources of Regional Data

The examples below use postcode boundary data available from the Australian Bureau of Statistics (the codes for performing this task are available in the Supplementary Material or on the website https://richardbeare.github.io/GeospatialStroke/). It is common for boundaries used in reporting of regional statistics to be available in standard file formats from the reporting bodies or central authorities along with the reported statistics. The regional demographics measures, often derived from national census data, also represent an important source of information for researchers, including age, sex, income, ethnicity etc. For example, in the US, key data sources on sociodemographics and the built environment include the Census Bureau's decennial census (18) (a complete enumeration at fine spatial scales but coarse, decadal temporal scales), American Community Survey (19) (a survey with annual temporal scales, but often fairly large standard errors at small spatial scales due to the sample size), and TIGER/Line shapefiles (20) of tract, municipal, and urbanized area boundaries. A comprehensive repository of US road network models at regional and municipal scales is available on the Harvard Dataverse (21). Additional regional data are frequently available from municipal, state, county, or metropolitan governmental agencies. Demographic data for countries in the European Union are provided by Eurostat (22). This includes time series data from several years to decades on economics, demography, infrastructure, health, traffic, and more of the EU (23). Geographic data for the EU is available through the Geographic Information System of the COmmission (GISCO), part of Eurostat. Similar levels of demographic data are available from France through INSEE (24), Germany through Destatis (25) and, Switzerland through (26). There are a number of European sources of geospatial data (27–29).

### 2.3. Geocoding and Reverse Geocoding

Location information, such as a patient's home address, is often available as a street address, rather than coordinates (a longitude/latitude pair). However, operations such as plotting addresses on a map require coordinates. Geocoding is the process of converting an address to a coordinate pair. Reverse geocoding converts a coordinate pair to an address. Coordinates are useful in many other types of computation, as we shall see in the examples below. There are two common approaches to geocoding and reverse geocoding. The most ubiquitous is via web services such as Google Maps. Other services, such as OpenStreetMap's Nominatim web service and OpenCage (https://opencagedata.com/), provide similar capabilities and all can be queried in an automated way from R and Python (30). The other approach is via a local database of geocoded addresses. One example, for Australia, is the PSMA (formerly Public Sector Mapping Agencies) address database available in an R queryable form. A local database allows many high speed queries, but is often less flexible in terms of query structure than the web services. Web services are discussed in more detail below.

### 2.4. Distance and Travel Time Estimation

A key part of a number of studies cited above is the estimation of travel time between patient and treatment center. The popularity of personal navigation systems in smartphones has driven the development of extremely sophisticated tools to estimate the fastest route between points. One of the best known, Google Maps^1^, uses a combination of information about the road network, historic travel time data derived from smartphone users and live information from smartphones. The travel time estimates are thus sensitive to time of day, weather conditions and possibly traffic accidents. Google, and other web services for travel time estimation, can be queried in a similar fashion to the geocoding services. It is also possible to create a local database to represent the road network, allowing more rapid querying, but losing some of the benefits of traffic models.

### 2.5. Visualization

Two forms of visualization are used in the following examples - static and interactive. Static maps are required for printed reports and typically present a carefully selected view. Interactive maps allow exploration of a data set, via zooming and toggling of overlays. Interactive maps often use web services to provide the background map “tiles,” over which data is superimposed. Different interactive web services specialize in different types of display. Some tools produce static and interactive displays in very similar ways.

### 2.6. Introduction to Web Services

Web services providing various forms of geospatial capabilities are a crucial component of the geospatial analysis tools now available to researchers. Web services deliver what used to be complex and specialized information products to the general public. Geocoding and travel time estimation are two common examples that have already been discussed. Other capabilities include delivery of tiled maps (such as the Google Maps display), street network and building footprint data (such as from OpenStreetMap), and census data on sociodemographic or built environment characteristics (such as from the US Census Bureau).

#### 2.6.1. Application Programming Interfaces (API)

Web services such as Google Maps are accessible via an API. The API allows software tools, such as *R* or *Python*, to make requests to the web service and retrieve results. Thus, if we consider the Google Maps example, not only can a user access a map query for an address via a web browser, but a program can submit the same request. Furthermore, a program can submit a series of automated requests. For example, given a list of addresses, it is relatively simple to generate an R or Python procedure to geocode all of them via a web service. Many APIs such as Google Maps are commercial products and thus charge for use, although the use is often free for small volumes (typical limit is 2,500 queries per day). The combination of these factors tends to mean that many APIs require somewhat complex setup, typically via signup and creation of keys. Terms of use may evolve over time, with charging being introduced, possibly leading to a need to enter credit card details (necessary for Google Maps API). We have endeavored to create examples that do not require keys, simplifying getting started (tmaptools and OpenStreetMap). However, some extensions have been included that do require keys. These are described in Supplementary Material.

#### 2.6.2. OpenStreetMap (OSM)

OSM (https://www.openstreetmap.org/) is a service collecting and distributing crowdsourced geospatial data. Many useful OSM services are available without API keys, and it is thus the platform of choice for examples in this paper. OSM is also unusual in that it allows access to underlying geospatial structures, such as road networks, rather than images generated from those structures. This capability is used to estimate travel time.

#### 2.6.3. Access to the Examples

The examples are available in their source code form from https://github.com/richardbeare/GeospatialStroke/archive/master.zip. “Live” versions and instructions are available at https://richardbeare.github.io/GeospatialStroke/index.html and can be viewed in conjunction with the methods section. The description focuses on the *R* versions of the examples. Code is visible in the shaded boxes, while output of the code, such as maps, are displayed immediately after the code. *Python* versions are provided and implement equivalent steps. Details on downloading and running the examples are available in Supplementary Material and at the web site.

## 3. Methods

The key sections of code used in the methods are presented and described in tables in this article. These pieces of code are not intended to be executed in isolation, and are best appreciated in the complete examples (both code and data), which are too long to include directly in the article, but are available for download, as described in section 2.6.3.

### 3.1. Example 1: Choropleth to Visualize Estimated Stroke Numbers

#### 3.1.1. Overview

We demonstrate accessing and using different data sources. The first is Australian Bureau of Statistics census data provided at the postcode level for population information, stratified by age, as well as postcode boundary information. The second data source is incidence data from the North East Melbourne Stroke Incidence Study (NEMESIS) (13). This is combined with the first dataset to estimate per-postcode stroke incidence. We demonstrate geocoding by finding the location of a hospital delivering acute stroke services, and then display postcodes within 15 km, coloring each postcode by estimated stroke incidence. The steps involved are described in Tables 1, 2.

**Table 1:**
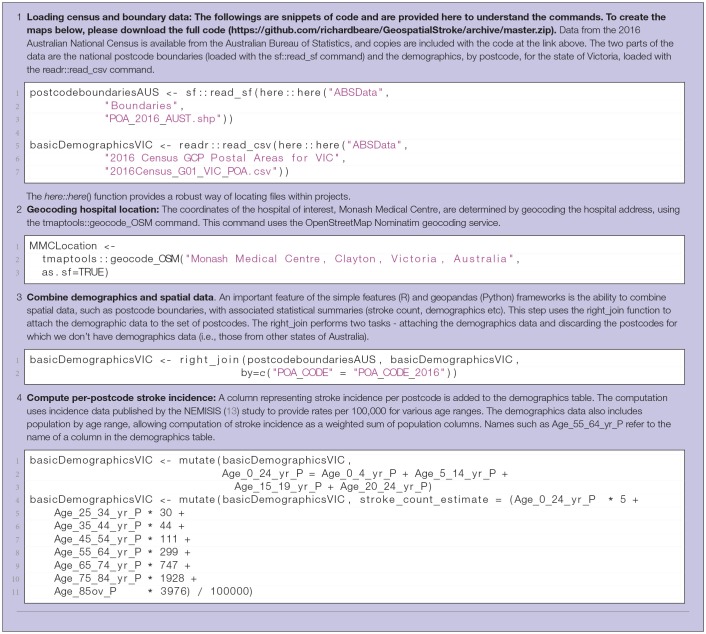
Steps 1-4 in computation of interactive display of choropleth of estimated stroke incidence. R code listings from the demonstration scripts is included.

**Table 2:**
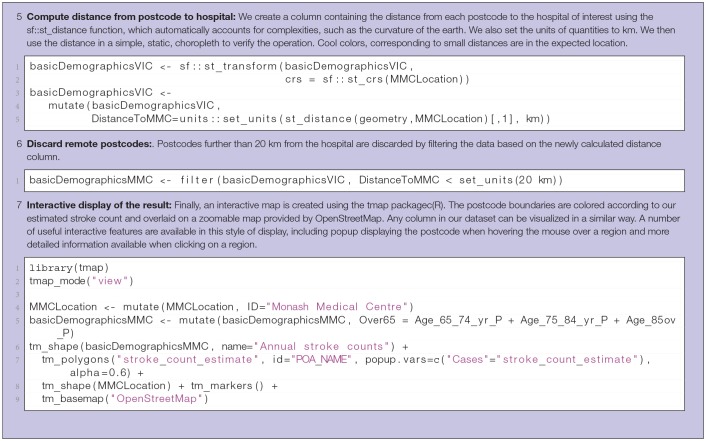
Steps 5-7 in computation of interactive display of choropleth of estimated stroke incidence.

#### 3.1.2. Example 2: Service Regions for Stroke Rehabilitation

In the second example we demonstrate the idea of estimating catchment basins for a set of three service centers (network comprising three rehabilitation hospitals servicing a hospital with an acute stroke service). The idea can be easily extended to more service centers. A catchment basin, or catchment area for a service center is the region that is closer to that service center than any other. The definition of “closer” is critical in this calculation, with travel time through the road network being a useful measure for many practical purposes. The approach used in this example involves the sampling of random addresses within a region of interest around the service centers, estimation of travel time from each address to each service center, assignment of addresses to the closest service center, combination of addresses based on service center to form catchment areas. The catchment areas can then be used to estimate loadings on service centers. The steps involved are described in Table 3.

**Table 3:**
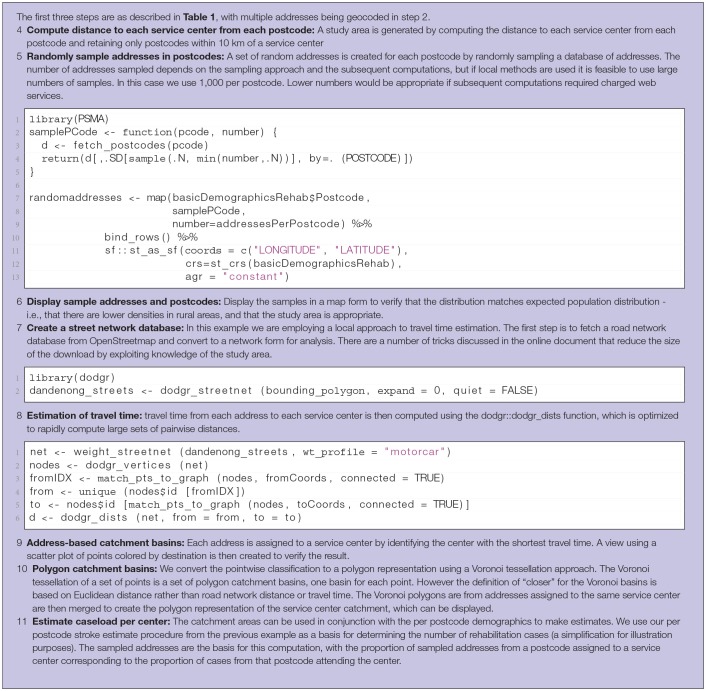
Steps in computation of catchment basin and case load for rehabilitation centers.

## 4. Results

The results of the two examples described above are a combination of source code and data and the output from the code. The output consists of visualizations of spatial data and estimates of rehabilitation loadings.

Complete versions of the examples are illustrated online at https://richardbeare.github.io/GeospatialStroke/index.html. A zipfile containing the complete data and code used to create the results can be downloaded from https://github.com/richardbeare/GeospatialStroke/archive/master.zip, and these two resources are the recommended starting point for readers interested in reproducing the results in this paper and getting started with experimentation with the ideas presented.

The key points of the examples are described in tables in the methods sections. However these points are best appreciated in the context of the complete examples.

### 4.1. Example 1: Choropleth to Visualize Estimated Stroke Numbers

**Figure 1:**
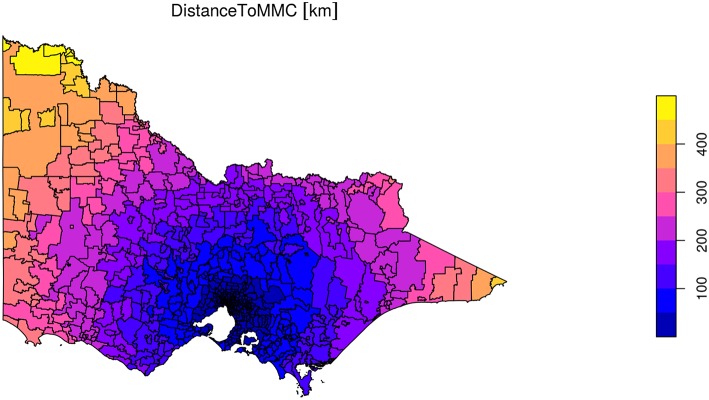
Visualization of straightline distance calculation from every postcode in the state to Monash Medical Centre. Static visualization created with *tmap*.

**Figure 2:**
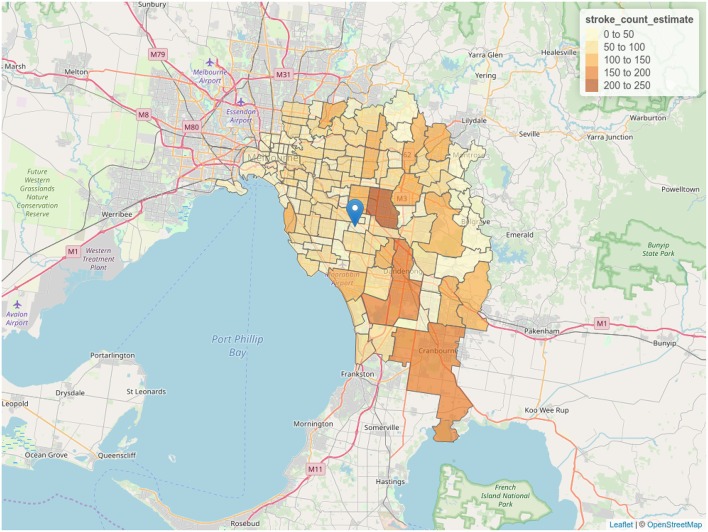
Choropleth of stroke case load estimate. Postcodes are colored according to estimation of stroke cases derived from demographic data. Screenshot of interactive visualization created with *tmap*.

Spatial data, as displayed by an *R* session, is illustrated in Tables S1, S2. Table S1 shows the hospital coordinates determined via geocoding, while a subset of the combined spatial, demographic and estimated stroke count data is illustrated in Table S2. A visualization of straight-line distance between each postcode and the hospital, useful for verifying the calculation is sensible, is illustrated in Figure 1. Finally, Figure 2 provides a screenshot of the interactive choropleth featuring postcodes in the vicinity of the hospital colored by estimated stroke case load. The display can be exported as a web page and viewed interactively, with the ability to pan and zoom and switch display layers on and off.

### 4.2. Example 2: Service Regions for Stroke Rehabilitation

**Figure 3:**
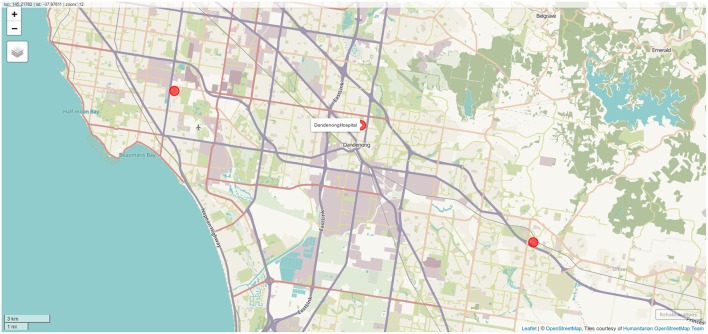
Locations of the three rehabilitation centers determined by geocoding. Screenshot of interactive visualization created with *mapview*.

**Figure 4:**
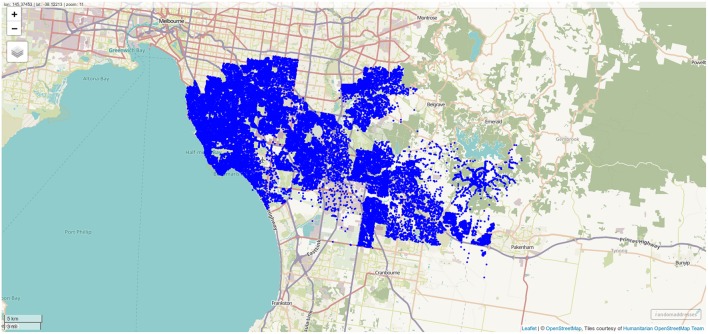
Randomly sampled addresses within the postcodes of interest. One thousand addresses per postcode were sampled, and the differing population density across the postcodes of interest is clearly visible. Screenshot of interactive visualization created with *mapview*.

**Figure 5:**
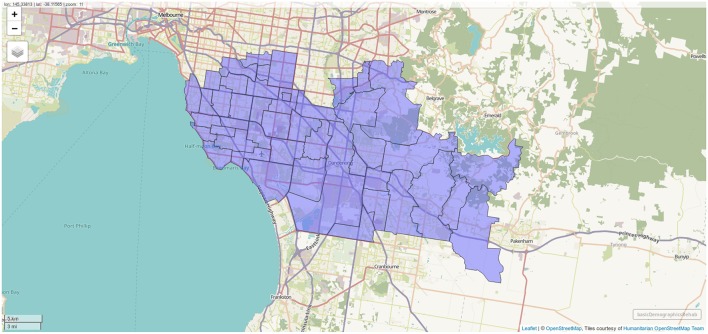
Boundaries of postcodes with centroids within 10 km of one of the rehabilitation centers. Screenshot of interactive visualization created with *mapview*.

**Figure 6:**
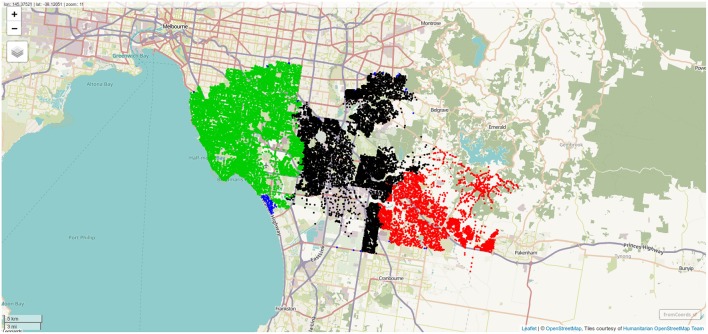
Sampled address colorcoded according to nearest destination, with distance to destination computed through the street network. Screenshot of interactive visualization created with *mapview*.

**Figure 7:**
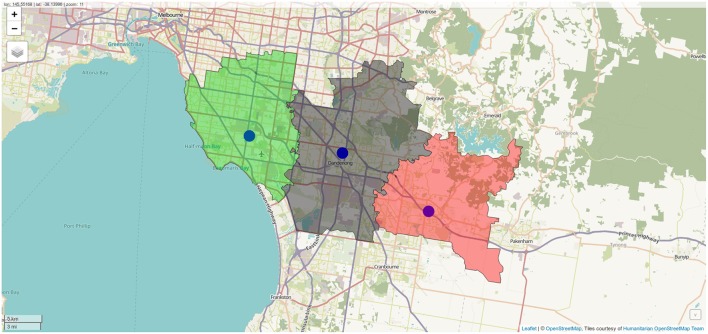
Polygon representation of rehabilitation center catchment zones. Screenshot of interactive visualization created with *mapview*.

**Figure 8:**
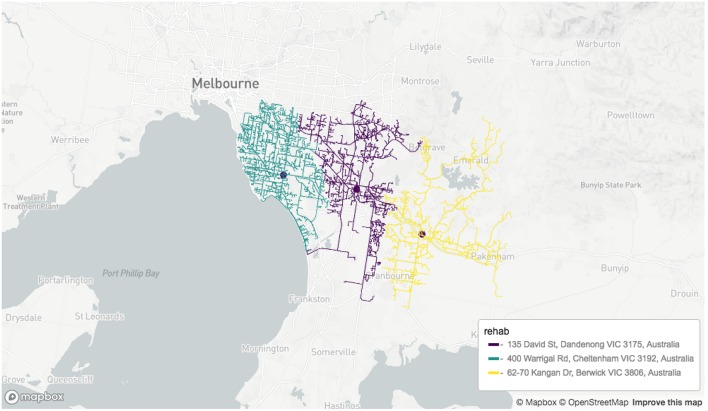
Road network-based visualization of catchment zones for rehabilitation centers. Screenshot of interactive visualization created with *mapdeck* (API key required).

**Figure 9:**
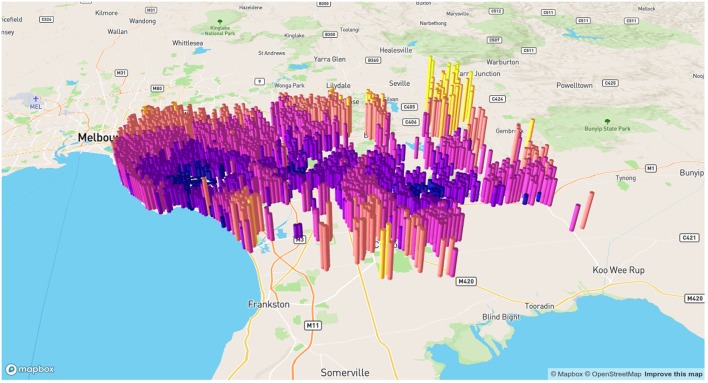
Alternative visualization of distances from random addresses to rehabilitation centeres. Screenshot of interactive visualization created with *mapdeck* (API key required).

**Figure 10:**
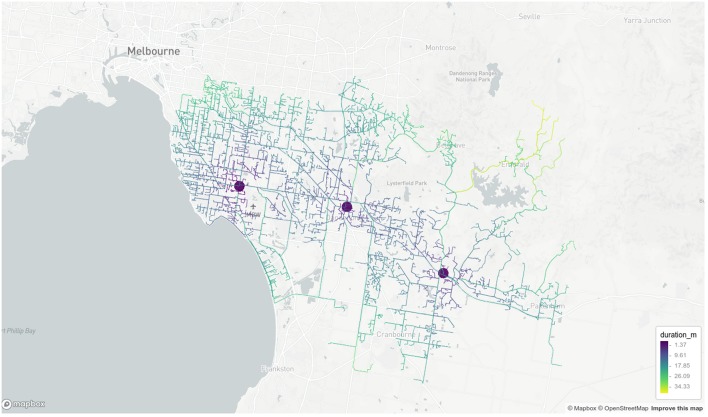
Road network visualization of travel times from random addresses to rehabilitation centeres. Screenshot of interactive visualization created with *mapdeck* (API key required).

Results of geocoding rehabilitation center addresses is shown in Table S3 with the corresponding visualization in Figure 3. A small selection of random addresses is available in Table S4 and the complete set is visualized in Figure 4. Boundaries of postcodes of interest are shown in Figure 5. Figures 6–8 show addressed-based, polygon-based and road-based views of the computed catchment basin. Figures 9, 10 show alternative visualizations of travel time using a hexagonal height map and a color coded road network. Finally, allocation of random addresses to rehabilitation centers and estimated case loads per catchment center are available in Tables 4, 5.

**Table 4:** The proportion of randomly sampled addresses allocated to each rehabilitation center via the distance calculation.

**Destination**	**Total**	**Percent**
CaseyHospital	6059	13.78
DandenongHospital	14184	32.26
KingstonHospital	23720	53.95

**Table 5:** The estimated case load per center, based on combination of per postcode prediction based on demographics and assignment based on distance calculations.

**Destination**	**Total**	**Percent**
CaseyHospital	289	10.67
DandenongHospital	882	32.55
KingstonHospital	1538	56.77

## 5. Discussion

There are many potential advantages to including geospatial variables such as location of patients and travel times to treatment centers when analyzing performance of, or modeling stroke treatment pathways. The ability to accurately model parts of the treatment pathway is important when fairly allocating limited resources, optimizing the placement of those resources, forecasting changes in loading in response to change in population distribution and investigating how to best utilize new technology or treatment options. In this article we have introduced some of the fundamental geospatial analysis components and provided reproducible examples using those components in a form that we hope will reduce the steep learning curve for researchers new to the area. In the writing of this paper, we heeded the call from this special issue of Frontiers in Neurology to provide source codes and enable both stroke researchers and “…citizen scientist to explore ideas in transportation and access to stroke therapy.” Recent years have seen increasing use of geospatial analysis techniques, similar to those explored in this paper, in stroke research as well as other health related areas. Two scenarios, with different spatial scales, for provision of acute stroke services are explored in Allen et al. (31) via an optimization framework, where the costs being optimized are derived from geospatial measures, including travel time. Implications of new technology (MSU) for rural patients is discussed in Mathur et al. (32) and urban application of the same technology is quantitatively analyzed in Phan et al. (33), with analysis exploiting travel time derived from web services to estimate deployment boundaries. Rather than providing further examples in acute stroke services of which are published in his special topic (31–33) we had provided examples here in terms of rehabilitation service provided by 3 hospitals attached to an ECR hub (Figures 3–10). Fair distribution of load across the hospital network is possible while allowing the patients and family unit in close contact, in keeping with the idea of patient centrad design. These studies discussed here were conducted to inform decisions under specific assumptions and in relation to individual urban environments (i.e. specific cities). However, they share common analytical ideas and data, namely geospatial analysis tools of the form explored in this paper. Use of these frameworks allow relevant research to be extended to different urban environments and extended as new assessment tools or treatment or transport options become available. For example, the tools described in this paper allow a researcher and citizen scientist to explore how the models relate to their own city as the key data, travel time and center location, can be collected easily from web services (10, 11) Furthermore, the analysis could be modified to investigate the effect of a new LVO assessment tool on decision making (34). The focus of discussion thus far has been on acute stroke services, however there are applications in many other health areas. For example, effects of traffic conditions on staff recall times for ST elevation myocardial infarction patients has been explored using similar approaches (35). Finally, geospatial analysis can be adapted to evaluation of brain health as seen in a recent study of the associations between brain measures and a neighborhood walkability index computed from geospatial data (36).

### 5.1. Limitations

In this project, we have provided examples in urban setting only. Modeling of transport in non-urban rural setting can be complex as it may have to take into account air transport.

A limitation of the current work in which the traffic model is based on Google Maps API, is that it does not take into account population growth and change in road network. In a paper in this journal, strategic transport model was used to assess stability of the geospatial model over time (37). Further geospatial analysis is only one component of a complex equation governing decisions on optimal centralized models for acute stroke therapy. As alluded to earlier in the article, the equation would also involve hospital capacity, availability of interventional neuroradiologists and stroke neurologists. For example, in the State of Victoria, Australia there are currently 13 certified interventional neuroradiologists with the majority of these currently in the two designated clot retrieval hubs http://www.ccinr.org.au/register (accessed 23/3/2019). As such adding a third hub in this centralized acute stroke therapy model would require acquisition of additional personnel. The choice of a model such as direct transfer to “mothership” for all patients would require modeling to estimate its effect on capacity of “mothership” to handle evaluation of all stroke cases since 84% of acute stroke cases do not go on to ECR (38). These types of modeling, such as discrete event simulation and agent-based modeling, were not covered in this introduction to software for geospatial analysis. An alternative strategy to support the “mothership” model is the use of bedside tools to screen patients for LVO (34). These tools have not yet been rigorously and prospectively tested in the field, nor has the impact of these tools on hospital case load been tested.

## 6. Conclusion

Computational frameworks facilitatinig analysis of geospatial data are now more accessible than ever before due to the combination of open source software tools, increasing availability of geospatial, demographic and other relevant health data from government and administrative bodies and a plethora of web services offering advanced geospatial data products. These tools are extremely powerful and flexible and offer the potential to address many important questions in stroke treatment. We hope this paper provides a useful introduction to researchers wanting to utilize spatial data. We invite the reader and citizen scientist to take the next step.

## Data Availability

Publicly available datasets were analyzed in this study. The data and code can be found here: https://github.com/richardbeare/GeospatialStroke/archive/master.zip.

## Author Contributions

RB, TP, MP, NT, DC, MS, and GB contributed to the research plan. RB, GB, MP, MS, and DC wrote the code. RB, TP, NT, GB, and MS wrote the manuscript and provided critical reviews.

### Conflict of Interest Statement

DC is employed by company Symbolix Pty Ltd. TP is on the Advisory Board of Genzyme on Fabry Disease and has received payment for lectures including service on speakers' bureaus for Bayer, Boehringer Ingelheim, Pfizer, and Genzyme. The remaining authors declare that the research was conducted in the absence of any commercial or financial relationships that could be construed as a potential conflict of interest.

## Footnotes

^1^the two APIs involved are the directions api and distance api.
