# Supplementary material for: An Introduction to Software Tools, Data, and Services for Geospatial Analysis of Stroke Services
Source: Front Neurol. 2019 Aug 7;10:743. doi: 10.3389/fneur.2019.00743 (PMC6693386; doi:10.3389/fneur.2019.00743)
Supplement: Supplementary file 1 [file Data_Sheet_1.pdf]

Supplementary Material

1 SUPPLEMENTARY MATERIAL

2 INTRODUCTION TO EXAMPLES

Supplementary material relating to this article is provided in the form of R and python source code and data that can be used to re-create the examples discussed in the text, as well as interactive versions of the outputs of those examples. The source material is available from Github.com and the live version is available here.

The article discusses two examples:

- Construction of a choropleth or thematic map.
- Estimation of catchment basins and case loadings for rehabilitation centres.

The package available for download (above) includes several implementations of these examples, as follows:

- *R* implementations of both that do not require installation of API keys: Choropleth, Catchment basins.
- *Python* implementations of both that do not require installation of API keys. Choropleth, Catchment basins.
- An alternative *R* implementation of the second example that utilises API keys to access Google services and Mapbox visualization services: Catchment basins with API keys.

The live versions of the examples requiring API keys do not include interactive visualizations. Examples must be recreated by the user, with their own keys, in order to use the visualization tools.

3 SOFTWARE SETUP

3.1 *R* setup

The R statistical environment can be obtained for Windows, Macintosh and Linux from www.r-project.org.

Rstudio, a modern graphical development environment for *R*, is also recommended and may be obtained from www.rstudio.com. The following instructions assume an installation of *Rstudio*.

The examples employ a number of *packages* to provide required functionality. These may be installed from inside *R* or *Rstudio* by issuing the following commands:

```
install.packages(c("tidyverse", "sf", "here", "units", "tmaptools",
                   "tmap", "knitr", "mapdeck", "googleway",
                   "mapview", "devtools", "dodgr", "viridisLite"))

devtools::install_github("HughParsonage/PSMA")
```

The interactive examples can then be created locally by opening the *R markdown* files, that have a *.Rmd* suffix, and click on the *knit* button in *Rstudio*.

3.2 *Python* setup

The miniconda tools are recommended for management of python installations. The commands below should be executed in the terminal (Mac/Linux) or the command prompt (Windows). The steps are:

1. Install miniconda
2. Change directory to the python folder:
   ```
   cd GeospatialStroke/Python
   ```
3. Create virtual environment:
   ```
   conda config --prepend channels conda-forge
   conda create -n GEO --strict-channel-priority --yes python=3 --file requir
   ```
   This command needs to be executed from within the python folder containing the *requirements.txt* file.
4. Activate virtual environment and install notebook kernel:
   ```
   conda activate GEO
   python -m ipykernel install --user --name GEO --display-name "Python (GEO)
   ```
5. Change directories in the terminal or command prompt to the location of the notebook folder and launch Jupyter to run the notebooks:
   ```
   jupyter lab
   ```

A *Jupyter notebook* server will run in your browser - select either *example1.pynb* or *example2.pynb* to open the examples.

In future sessions on the following commands are needed to start the notebook:

```
conda activate GEO
jupyter lab
```

3.3 API Keys and tokens

Online services which offer an interface to their applications will sometimes require use of an API key, or application programming interface key. This key should be unique for each user, developer or application making use of the service as it is a way for the provider to monitor and, where applicable, charge for use.

Two major mapping platforms that require an API key are Google Maps and Mapbox, both of which are used in the second version of the catchment basin example. At the time of writing both allow unrestricted use of the mapping API. However, Google has limits on the other services it offers such as geocoding and direction services.

Both Google and Mapbox require users create an account.

The required Google API keys may be obtained by following instructions provided by Google.

The required Mapbox token may be obtained by following instructions provided by Mapbox.

4 SUPPLEMENTARY TABLES

```
Simple feature collection with 1 feature and 7 fields
geometry type:  POINT
dimension:      XY
bbox:           xmin: 145.1207 ymin: -37.92093 xmax: 145.1207 ymax: -37.92093
epsg (SRID):    4326
proj4string:    +proj=longlat +datum=WGS84 +no_defs
                                                  query       lat      lon   lat_min   lat_max
1 Monash Medical Centre, Clayton, Victoria, Australia -37.92093 145.1207 -37.92098 -37.92088
   lon_min  lon_max                geometry
1 145.1207 145.1208 POINT (145.1207 -37.92093)
```

Table S1. Geocoding results for emergency hospital (Monash Medical Center).

```
Simple feature collection with 6 features and 4 fields
geometry type:  MULTIPOLYGON
dimension:      XY
bbox:           xmin: 144.9055 ymin: -37.85553 xmax: 144.9914 ymax: -37.79821
epsg (SRID):    4326
proj4string:    +proj=longlat +datum=WGS84 +no_defs
# A tibble: 6 x 5
  POA_NAME Tot_P_P stroke_count_est... DistanceToMMC                            geometry
  <chr>      <int>             <dbl>          [km]                    <MULTIPOLYGON []>
1 3000       37975            24.7        15.77496 (((144.9576 -37.79972, 144.9588 -37
2 3002        4964            16.8        15.87279 (((144.9732 -37.80792, 144.9826 -37
3 3003        5515             5.14       18.86105 (((144.9165 -37.79821, 144.9257 -37
4 3004        9307            28.1        14.13294 (((144.985 -37.84569, 144.9842 -37
5 3005         525             0.578      18.11235 (((144.9479 -37.82339, 144.948 -37.
6 3006       18808            20.5        16.57805 (((144.956 -37.82305, 144.9579 -37.
```

Table S2. Subset of simple features (sf) table containing both demographic and postcode boundary information for postcodes within 20km of the emergency service center. Colums displayed are postcode name, total population, estimate number of stroke cases, distance to emergency center and the postcode geometry. The estimate of stroke cases was based on a combination of population age bands (not illustrated) and incidence data from the NEMISIS study. The distance column was computed between the geometry column of this table ant he geometry column of the geocoded hospital locaiton using the *sf::st_distance* function.

```
Simple feature collection with 3 features and 7 fields
geometry type:  POINT
dimension:      XY
bbox:           xmin: 145.0797 ymin: -38.04446 xmax: 145.3457 ymax: -37.95604
epsg (SRID):    4283
proj4string:    +proj=longlat +ellps=GRS80 +towgs84=0,0,0,0,0,0,0 +no_defs
                                                                   query       lat      lon   lat_min   lat_max
DandenongHospital   Dandenong Hospital, Dandenong VIC 3175, Australia -37.97611 145.2178 -37.97728 -37.97545
CaseyHospital             62-70 Kangan Dr, Berwick VIC 3806, Australia -38.04446 145.3457 -38.04539 -38.04446
KingstonHospital  The Kingston Centre, Heatherton VIC 3202, Australia -37.95604 145.0797 -37.95830 -37.95344
                   lon_min  lon_max                   geometry
DandenongHospital 145.2162 145.2198 POINT (145.2178 -37.97611)
CaseyHospital     145.3456 145.3457 POINT (145.3457 -38.04446)
KingstonHospital  145.0768 145.0810 POINT (145.0797 -37.95604)
```

Table S3. Geocoded locations for the 3 rehabilitation centers.

```
Simple feature collection with 6 features and 13 fields
Attribute-geometry relationship: 13 constant, 0 aggregate, 0 identity
geometry type:  POINT
dimension:      XY
bbox:           xmin: 145.0398 ymin: -37.89162 xmax: 145.0865 ymax: -37.86661
epsg (SRID):    4283
proj4string:    +proj=longlat +ellps=GRS80 +towgs84=0,0,0,0,0,0,0 +no_defs
  POSTCODE ADDRESS_DETAIL_INTRNL_ID STREET_LOCALITY_INTRNL_ID BUILDING_NAME LOT_NUMBER FLAT_NUMBER
1     3145                 11867025                    590471          <NA>       <NA>         126
2     3145                 10017734                    530005          <NA>       <NA>          10
3     3145                 10204296                    526751          <NA>       <NA>          NA
4     3145                 11223826                    528755          <NA>       <NA>          NA
5     3145                  9964136                    473522          <NA>       <NA>           5
6     3145                  9950516                    421545          <NA>       <NA>          NA
  NUMBER_FIRST STREET_NAME STREET_TYPE_CODE lat_int  lat_rem lon_int lon_rem                  geometry
1         1341   DANDENONG             ROAD     -37 -8876953     145  805995  POINT (145.0806 -37.8877)
2           13      LLOYDS           AVENUE     -37 -8857273     145  445100 POINT (145.0445 -37.88573)
3           94    TOORONGA             ROAD     -37 -8666070     145  398309 POINT (145.0398 -37.86661)
4          368    WAVERLEY             ROAD     -37 -8775029     145  594729  POINT (145.0595 -37.8775)
5           18       CAPON           STREET     -37 -8872378     145  865195 POINT (145.0865 -37.88724)
6            6      CARRUM           STREET     -37 -8916150     145  863720 POINT (145.0864 -37.89162)
```

Table S4. Randomly sampled addresses from the PSMA data base.
